# The Protective Effect of Topical Spermidine on Dry Eye Disease with Retinal Damage Induced by Diesel Particulate Matter2.5

**DOI:** 10.3390/pharmaceutics13091439

**Published:** 2021-09-10

**Authors:** Hyesook Lee, Da Hye Kim, Hyun Hwangbo, So Young Kim, Seon Yeong Ji, Min Yeong Kim, Jung-Hyun Shim, Sun-Hee Leem, Jin Won Hyun, Gi-Young Kim, Yung Hyun Choi

**Affiliations:** 1 Department of Biochemistry, College of Korean Medicine, Dong-Eui University, Busan 47227, Korea; 14769@deu.ac.kr (H.L.); believe0402@pusan.ac.kr (D.H.K.); hbhyun2002@pusan.ac.kr (H.H.); 14731@deu.ac.kr (S.Y.K.); 14602@deu.ac.kr (S.Y.J.); ilytoo365@deu.ac.kr (M.Y.K.); 2 Anti-Aging Research Center, Dong-Eui University, Busan 47340, Korea; 3 Department of Pharmacy, College of Pharmacy, Mokpo National University, Mokpo 58554, Korea; s1004jh@mokpo.ac.kr; 4 Department of Biomedical Sciences, Dong-A University, Busan 49315, Korea; shleem@dau.ac.kr; 5 Department of Health Sciences, Dong-A University, Busan 49315, Korea; 6 Department of Biochemistry, College of Medicine, Jeju National University, Jeju 63243, Korea; jinwonh@jejunu.ac.kr; 7 Department of Marine Life Science, Jeju National University, Jeju 63243, Korea; immunkim@jejunu.ac.kr

**Keywords:** dry eye disease, inflammation, lacrimal gland, spermidine, particulate matter2.5

## Abstract

Air pollutants, especially ambient fine particulate matter2.5, may contribute to various ocular surface disorders, including dry eye disease, keratitis and conjunctivitis. A natural polyamine spermidine has a protective effect on the retina and optic nerve; however, no study has been conducted on the application of spermidine in particulate matter2.5-induced dry eye disease. In the present study, we investigated the effect of spermidine eye drops in topically exposed particulate matter2.5-induced dry eye models of Sprague-Dawley rats, by hematological, biochemical and histological evaluation. Spermidine eye drops attenuated the particulate matter2.5 exposure-induced reduction of tear secretion and corneal epithelial damage. Furthermore, spermidine protected against conjunctival goblet cell loss and retinal ganglion cell loss induced by particulate matter2.5. Additionally, spermidine markedly prevented particulate matter2.5-induced infiltration of cluster of differentiation3^+^ and cluster of differentiation4^+^ T lymphocytes and F4/80^+^ macrophages on lacrimal gland. Moreover, over expression of pro-inflammatory cytokines, including tumor necrosis factor-α, interleukin-6 and interleukin-17 in the lacrimal gland and cornea. Meanwhile, the levels of serum total cholesterol and low-density lipoprotein cholesterol were markedly increased by topical exposure to particulate matter2.5, but this change in the lipid profile was decreased by spermidine. Taken together, spermidine may have protective effects against particulate matter2.5-induced dry eye symptoms via stabilization of the tear film and suppression of inflammation and may in part contribute to improving retinal function and lipid metabolism disorder.

## 1. Introduction

The World Health Organization (WHO) recently announced that nine out of 10 people breathe air that exceeds the WHO guideline limits containing high levels of pollutants, and 4.2 million deaths occur annually as a result of exposure to ambient air pollution [1,2]. Air pollution is a heterogeneous mixture of various biologically toxic substances that cause detrimental health outcomes [3,4]. Among them, fine particulate matter (aerodynamic diameter below 2.5 μm, PM_2.5_) is considered the most important air quality indicator and is a great harmful substrate of health threat [5]. Recently accumulated epidemiological and biochemical evidence has shown that there has been an upsurge of interest in the harmful effects of PM_2.5_ on the eyes, which is one of the several organs that are directly exposed to the external environment [6,7,8]. Epidemiological studies have reported that chronic or acute exposure to PM_2.5_ may be attributed to various ocular surface disorders, including dry eye disease (DED), keratitis and conjunctivitis, accompanied by symptoms such as foreign body sensation, redness, stinging, pain and burning [9,10,11]. In addition, several animal studies have demonstrated that topical administration of PM_2.5_ results in tear production decrease, tear film destruction, mucin secretion repression and inflammation of the lacrimal gland in mice [12,13,14]. These ocular changes are similar to the symptoms of dry eye in humans [12]. More recently, a few studies have suggested that exposure to PM_2.5_ promotes cytotoxicity, cellular organelle damage, inflammation and wound healing suppression in corneal epithelial and conjunctival epithelial cells [15,16,17]. Nevertheless, most studies on the biological and toxicological risks of PM_2.5_ have focused on other organs, such as the cardiovascular and respiratory systems, while studies on the influence of PM_2.5_ on the ocular system are still in the early stage.

Natural polyamines are essential for biological processes, including cell growth, proliferation and differentiation [18]. Spermidine, a polyamine, is mostly accumulated in the brain and retina, and acts as an endogenous free radical scavenger and a potential inhibitor of reactive oxygen species (ROS) [19,20,21]. Spermidine has been reported to attenuate the production of pro-inflammatory cytokines in lipopolysaccharide-stimulated microglia [22] and inhibit oxidative damage in hydrogen peroxide (H_2_O_2_)-treated fibroblasts [20]. Furthermore, it has been demonstrated that spermidine attenuated autoimmune encephalomyelitis, a model for multiple sclerosis (a chronic autoimmune disease of the nervous system), through the inhibition of macrophage inflammation [23]. Noro et al. [21] reported that oral administration of spermidine has a protective effect on retinal ganglion cell damage and optic nerve regeneration in a mouse with optic nerve injury. In addition, they demonstrated the efficacy of spermidine in suppressing neurodegeneration in a normal tension glaucoma mouse model [24]. Moreover, Guo et al. [25] suggested that spermidine alleviates H_2_O_2_-mediated retinal ganglion cell death and prevents optic nerve demyelination in autoimmune encephalomyelitis mice. Previously, we demonstrated that spermidine suppressed H_2_O_2_-induced apoptosis in retinal pigment epithelial cells by blocking calcium overload [26]. Even though spermidine has a protective effect on the retina and optic nerve, the effect of topical administration of spermidine on the ocular system has not been sufficiently elucidated. In addition, to our best knowledge, no study has been conducted on the application of spermidine in PM_2.5_ -induced ocular disease. Therefore, in this study, we investigated the effect of spermidine eye drops on changes in the eye, including the cornea, conjunctiva, lacrimal gland and retina, and on the changes in hematology and biochemistry in dry eye rat models induced by topical exposure to PM_2.5_.

## 2. Materials and Methods

### 2.1. Preparation of PM_2.5_ and Eye Drops

The National Institute of Standards and Technology (NIST, Gaithersburg, MD, USA) SRM 1650b standard diesel PM_2.5_ and spermidine were purchased from Sigma-Aldrich Chemical Co. (St. Louis, MO, USA). PM_2.5_ was dissolved in normal saline (JW Pharmaceutical, Seoul, Korea) to prepare 5 mg/mL eye drop solution. The following were prepared and dissolved in normal saline: 5 mg/mL PM_2.5_, 0.2% spermidine and 0.5% spermidine drops. Topical CsA (0.05% Cyporin N^®^ eye drops) was obtained from Taejoon Pharma Co., Ltd. (Seoul, Korea).

### 2.2. Animals and Experimental Procedures

Animal care and all experiments were performed in accordance with the Guide for Animal Experimentation of Dong-eui University with the approval of the Institutional Animal Care and Use Committee (IACUC approval No. R2019-005; date of approval 15 May 2019). Six-week-old female Sprague-Dawley (SD) rats were obtained from Samtako Bio Korea (Osan, Korea) and housed in a semi-pathogen-free facility with a temperature of 22–24 °C, relative humidity of 50–60% and 12 h light/12 h dark cycles. After acclimatization for a week, the rats were randomly divided into five groups: (1) Normal group (normal, *n* = 5); (2) PM_2.5_ vehicle group (vehicle, *n* = 5); (3) PM_2.5_ with 0.2% spermidine-treated group (0.2% SP, *n* = 5); (4) PM_2.5_ with 0.5% spermidine-treated group (0.5% SP, *n* = 5); and PM_2.5_ treated with CsA (*n* = 5). DED was induced by topically administering 20 μL of 5 mg/mL PM_2.5_ in both eyes four times daily for 14 days except for the normal group. During the same period, all eye drops were administered 30 min after PM_2.5_ exposure. In brief, 20 μL of 0.2% or 0.5% spermidine was administered in both eyes of rats in the spermidine-treated rats, four times daily. All eyes of rats in the CsA-treated group were topically exposed to 20 μL of CsA, twice daily for 14 days. Both eyes of rats in the PM_2.5_ vehicle group received 20 μL of normal saline, which was used as a vehicle solution, four times daily. Body weight was measured at baseline and on day 14. The rats in all the groups were euthanized on day 14. Whole blood was collected directly from the heart, placed in heparinized tubes and allowed to clot for 30 min at room temperature. Thereafter, the blood was centrifuged at 3000 rpm for 10 min at 4 °C to obtain serum, which was stored at −80 °C for subsequent analysis, as previously described [27]. After perfusion, the organs were immediately excised and weighed. The eyes and adnexa were dissected and fixed in 10% formalin for histological and immunohistochemical analyses.

### 2.3. Hematological and Biochemical Analysis

Hematological analysis was determined using a Sysmex XN-9000 analyzer (Sysmex Corporation, Kobe, Japan). Serum alanine aminotransferase, aspartate aminotransferase, alkaline phosphatase, blood urea nitrogen, creatinine and lipid profiles were analyzed using Cobas 8000 C702 chemistry analyzer (Roche, Mannheim, Germany).

### 2.4. Tear Production

Tear volume was measured using phenol red tear threads (Jingming Ltd., Tianjin, China) on days 0, 7 and 14 post-treatment, as previously described [28]. Briefly, threads were inserted into the lateral canthus of the lower eyelid for 1 min. The length of the red portion of the threads was measured, and the tear volume was expressed in millimeters (mm).

### 2.5. Corneal Fluorescein Staining (CFS)

Fluorescein staining was performed by adding 1% sodium fluorescein (Sigma-Aldrich Chemical Co.). The eyes were observed with an SL-D7 slit-lamp biomicroscope (Topcon Medical Systems, Inc., Oakland, NJ, USA) under cobalt blue light. The fluorescein score was analyzed using a standardized National Eye Institute grading system on captured images from 0 to 3 points in five areas of the ocular surface [29].

### 2.6. Hematoxylin and Eosin (H&E) Staining

Fixed eyeballs and lacrimal glands were embedded in paraffin and cut into 5-μm sections using a microtome (Leica RM2245, Leica Biosystems, Heidelberg, Germany). The sections were deparaffinized, hydrated and stained with hematoxylin (YD Diagnostics Co., Yongin, Korea) for 3 min. Subsequently, the slides were washed with hydrochloride solution and stained with eosin (YD Diagnostics Co.) for 1 min. The stained slides were dehydrated, mounted and observed using the EVOS FL Auto 2 imaging system (Thermo Fisher Scientific, Waltham, MA, USA).

### 2.7. Periodic Acid-Schiff (PAS) Staining

The entire globe, including forniceal conjunctiva, was embedded in paraffin, cut into 5-μm thick sections and stained using a PAS kit (Sigma-Aldrich Chemical Co.) according to the manufacturer’s protocol. Images of violet PAS-positive goblet cells were acquired using a microscope (Carl Zeiss, Oberkochen, Germany) at the Core Facility Center for Tissue Regeneration (Dong-eui University, Busan, Korea), and goblet cell density was determined by counting the PAS-positive cells of 100 μm^2^ in five different sections.

### 2.8. Immunohistochemistry

For immunohistochemical analysis, 5-μm thick sections of the lacrimal gland and eyeball were deparaffinized, hydrated, processed in antigen retrieval solution (Abcam, Inc., Cambridge, UK) and exposed to 3% H_2_O_2_ solution (Sigma-Aldrich Chemical Co.) for 30 min. The slides were incubated with primary antibodies against cluster of differentiation 3 (CD3, Abcam, Inc.), CD4 (Novus Biologicals, Littleton, CO, USA), F4/80 (Abcam, Inc.), interleukin-6 (IL-6, Abcam, Inc.), interleukin-17 (IL-17; Abcam, Inc.) and tumor necrosis factor alpha (TNF-α; Abcam, Inc.) for 1 h. Subsequently, the sections were incubated with secondary antibodies (DAKO Corp, Glostrup, Denmark) for 40 min, followed by probing with diaminobenzidine chromogen, and counterstained with Mayer’s hematoxylin (YD Diagnostics Co.). The stained slides were photographed using an imaging system (Thermo Fisher Scientific). The quantitative analysis of histological staining performed using “threshold tool” of ImageJ^®^ (National Institutes of Health, Bethesda, MD, USA).

### 2.9. Statistical Analysis

Data are presented as the mean ± standard deviation. The data of tear production and CFS were analyzed using SPSS version 22.0 (SPSS Inc., Chicago, IL, USA) and two-way ANOVA was used for statistical analysis. To conduct for analysis of other parameters, one-way analysis of ANOVA and post-hoc analyses were performed for comparisons between groups using GraphPad Prism 5.03 (2010, GraphPad Software Inc., La Jolla, CA, USA). Statistical significance was set at *p* < 0.05.

## 3. Results

### 3.1. Changes in Physiological Condition after 2 Weeks of Treatment in PM_2.5_-Topical Exposed Sprague-Dawley (SD) Rats

Body weight was measured in all groups at 0 and 14 days after topical exposure to PM_2.5_, with or without treatment. As shown in Table 1, no significant differences were observed in body weight gain between the normal group and PM_2.5_-exposed groups. Furthermore, the weights of the selected organs were not significantly different between the normal group and PM_2.5_-exposed groups.

### 3.2. Effect of Spermidine on the Changes of Hematological, Biochemical and Lipid Profiles in PM2.5-Topical Exposed SD Rats

The hematological analysis results, including red blood cell (RBC) count, white blood cells (WBC) count, hematocrit, hemoglobin levels, mean corpuscular volume (MCV), mean corpuscular hemoglobin (MCH) levels and platelet count, showed no differences among the groups (Table 2). In addition, no biochemical abnormalities were observed among the groups. However, the levels of total cholesterol (TC) were markedly increased to 74.42 mg/dL following topical exposure to PM_2.5_, and the levels significantly decreased to normal levels after topical administration of 0.5% spermidine, which was similar to the levels observed after CsA administration. Moreover, low-density lipoprotein cholesterol (LDL-C) levels were also significantly upregulated in the PM_2.5_ vehicle group, and the levels were downregulated following 0.2% and 0.5% spermidine administration, but there was no statistically significant difference compared with the vehicle group. Meanwhile, the levels of high-density lipoprotein cholesterol, triglyceride and free fatty acid were not different among the groups.

### 3.3. Effect of Spermidine on Tear Secretion and CFS after Topical Exposure to PM_2.5_ in SD Rats

**Figure 1:**
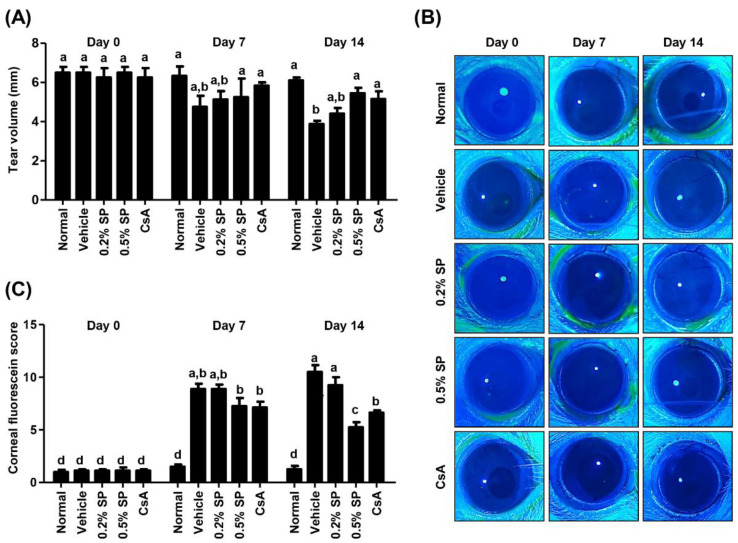
Effect of spermidine on tear secretion and corneal fluorescein staining (CFS) after topical exposure to PM_2.5_ in SD rats. (**A**) Changes in tear volume. The volume of tears secreted is measured using the phenol red thread test. The length of the thread that changed color to red is shown for each group at days 0, 7 and 14. (**B**) Representative images of CFS. (**C**) Changes in CFS score at days 0, 7 and 14. The data are expressed as the means ± standard deviation (*n* = 5). ^a^ Bars are significantly different at *p* < 0.05 from Bars with ^b^, ^c^, and ^d^. ^b^ Bars are significantly different at *p* < 0.05 from Bars with ^a^, ^c^, and ^d^. ^c^ Bars are significantly different at *p* < 0.05 from Bars with ^a^, ^b^, and ^d^. ^d^ Bars are significantly different at *p* < 0.05 from Bars with ^a^, ^b^, and ^c^.

We evaluated the effect of the topical administration of spermidine on the changes in tear production and CFS scores in PM_2.5_-applied SD rats. On days 0, 7 and 14, the tear volume and CFS score were measured using phenol red tear threads and sodium fluorescein staining, respectively. At day 0, no significant difference was observed in tear production and CFS score among all groups (Figure 1A–C). During the entire study period, the normal group showed no significant difference in tear production and CFS scores. At day 7, tear volume was markedly decreased in the PM_2.5_-treated vehicle group (4.75 ± 1.96 mm) compared with that in the normal group (6.33 ± 1.77 mm, Figure 1A). Even though the PM_2.5_ with 0.2% spermidine-treated group (5.13 ± 0.85 mm) and the PM_2.5_ with 0.5% spermidine-treated group (5.25 ± 1.89 mm) showed no significant difference in tear volume compared with the vehicle group, the tear production gradually increased in the 0.5% SP group. At day 14, PM_2.5_ treatment greatly suppressed tear secretion to 3.88 ± 0.57 mm, but topical administration of 0.5% spermidine markedly improved tear production (5.45 ± 0.86 mm) similar to normal levels. Meanwhile, tear production in the CsA group was significantly increased compared to that in the vehicle group over the entire time period. As shown in Figure 1B,C, the CFS score was significantly increased to approximately 8.03 ± 1.89 by topical application of PM_2.5_ for 7 days, and the levels showed no significant difference among all groups except the normal group. At day 14, the CSF score was more increased by PM_2.5_ application, whereas the CFS scores were markedly decreased compared with vehicle group to 5.25 ± 0.96 and 6.63 ± 0.48 in 0.5% SP and CsA groups, respectively. These results showed that topical exposure to PM_2.5_ induced tear film instability and ocular surface damage, whereas PM_2.5_-mediated damage to the tear film and cornea was markedly restored by spermidine application.

### 3.4. Effect of Spermidine on Detachment of Corneal Epithelium in a Rat Model of PM_2.5_-Induced DED

**Figure 2:**
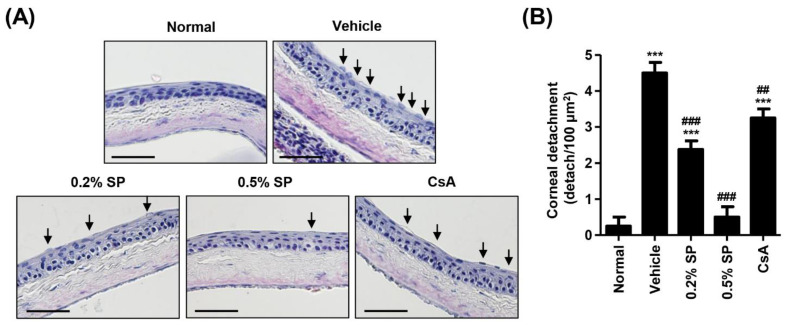
Effect of spermidine on the detachment of corneal epithelium in a rat model of PM_2.5_-induced dry eye diseases (DED). (**A**) Representative images of H&E-stained images of corneal sections. Black arrows indicate the detached and swollen epithelium. Scale bar; 50 μm. (**B**) The numbers of the detached corneal epithelium of 100 μm^2^ in five different sections were counted. Each bar indicates mean ± standard deviation (*n* = 5). The statistical analyses were conducted using analysis of ANOVA-Tukey’s post hoc test between groups. *** *p* < 0.001 compared to the normal group. ^##^ *p* < 0.01 and ^###^ *p* < 0.001 compared to the vehicle group.

To investigate whether topical application of PM_2.5_ changes the corneal epithelium and the effect of spermidine on PM_2.5_-mediated epithelium alteration, we performed H&E staining. Figure 2A shows that the detachment and swelling of corneal epithelium were more frequently observed in the vehicle group; however, that alteration following exposure to PM_2.5_ exposure was greatly reduced in the 0.2% SP, 0.5% SP and CsA groups. As shown in Figure 2B, the quantitative levels of the detached epithelium are indicated as number per 100 μm^2^. Topical exposure to PM_2.5_ significantly increased the detachment of corneal epithelium to 4.50 ± 0.58/100 μm^2^. However, this value by PM_2.5_ was markedly decreased to 2.38 ± 0.48/100 μm^2^ in the 0.2% SP group. Additionally, the detachment of corneal epithelium in the 0.5% SP group was greatly suppressed to 0.50 ± 0.58/100 μm^2^ compared with that in the vehicle group. This result indicated that the detachment of corneal epithelium following topical exposure to PM_2.5_ significantly decreased after topical administration of 0.5% spermidine, and its efficacy was superior to that of CsA.

### 3.5. Effect of Spermidine on Conjunctival Goblet Cell Population in a Rat Model of PM_2.5_-Induced DED

**Figure 3:**
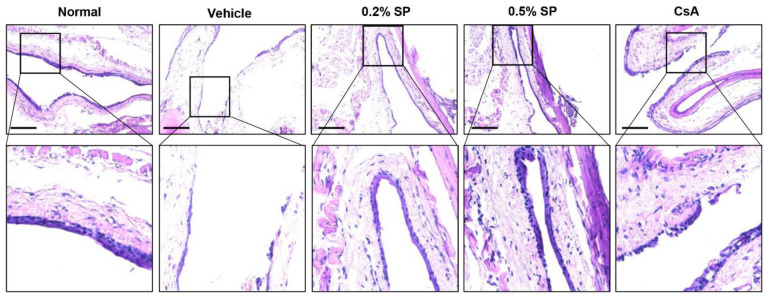
Effect of oral administration of CFW on conjunctival fornix goblet cell population in a rat model of PM_2.5_-induced DED. Representative images of PAS-stained images of conjunctival fornix sections. The goblet cells that stained a strong violet color. Scale bar; 100 μm.

To evaluate the population of goblet cells that secrete gel-forming mucins in the conjunctiva, we performed PAS staining. In normal rats, a large number of violet PAS-positive goblet cells were observed in the conjunctival fornix tissue (Figure 3). However, topical exposure to PM_2.5_ greatly suppressed the frequency of PAS-stained goblet cells. Nevertheless, PM_2.5_-mediated conjunctival goblet cell loss was substantially improved by topical eye drops of spermidine in a dose-dependent manner. In addition, the population of conjunctival goblet cells in the 0.5% SP group was higher than that in the CsA group. This result suggested that conjunctival goblet cell loss, a DED-mediated event, was markedly induced following exposure to PM_2.5_, and this alteration was significantly improved by topical administration of spermidine.

### 3.6. Effect of Spermidine on Inflammation of Lacrimal Gland and Cornea in a Rat Model of PM_2.5_-Induced DED

**Figure 4:**
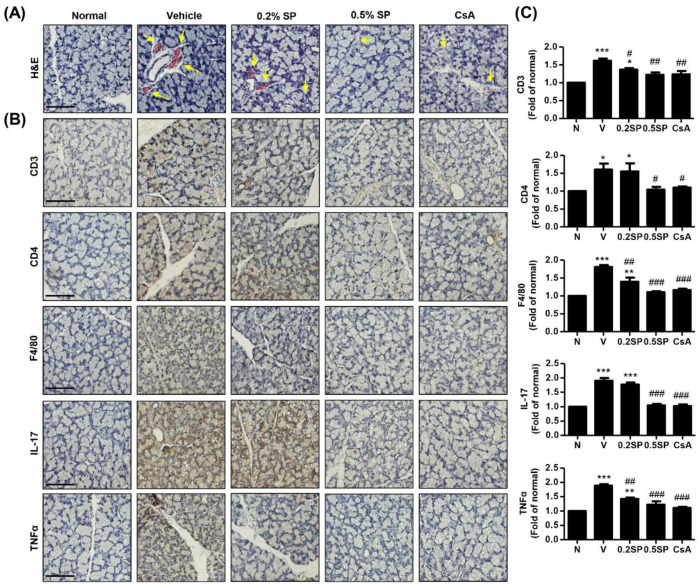
Effect of spermidine on inflammation of the lacrimal gland in a rat model of PM_2.5_-induced DED. (**A**) Representative images of H&E staining in the lacrimal gland. Yellow arrows indicate neo-vessels. Scale bar; 50 μm. (**B**) Representative images of immunohistochemical staining for CD3, CD4, F4/80, IL-17 and TNF-α, in lacrimal gland sections. Scale bar; 50 μm. (**C**) The stained area of the photograph was quantitative analyzed using ImageJ^®^ and calculated in terms of the fold of the normal. The data are expressed as the means ± standard deviation (*n* = 3). * *p* < 0.05, ** *p* < 0.01 and *** *p* < 0.001 compared to normal group. ^#^ *p* < 0.05, ^##^ *p* < 0.01 and ^###^ *p* < 0.001 compared to the vehicle group.

We assessed the effect of spermidine on the pathological changes in the lacrimal gland in rats with DED following exposure to PM_2.5_. Figure 4A shows normal secretory gland histology, including tight acini and ducts in normal rats. However, exposure to PM_2.5_ led to an increase in inflammatory cell infiltration, sizable interstitial edema with abnormal acini, and formation of neo-vessels around lobules. In contrast, topical administration of 0.5% spermidine markedly decreased PM_2.5_-mediated histopathological changes in the lacrimal gland. Meanwhile, cyclosporine administration also inhibited infiltration of inflammatory cells and edema with abnormal acini, but neo-vessels around lobules were still slightly present. Furthermore, to establish which type of cells were infiltrated, immunohistochemistry was performed the expression of CD3, CD4 and F4/80 on infiltrating cells. As shown in Figure 4B, many CD3^+^ T lymphocytes, CD4+ T lymphocytes and F4/80^+^ macrophages were observed surrounding the lesions on the sections of lacrimal gland obtained from PM_2.5_-exoposed rats. Up-regulated expression of CD3, CD4 and F4/80 by PM_2.5_ exposure was markedly down-regulated by 0.5% spermidine and cyclosporine treatments (Figure 4B,C). In addition, PM_2.5_ topical exposure greatly enhanced the expression of pro-inflammatory cytokines, such as IL-17 and TNF-α in the lesions of the lacrimal gland, whereas the upregulated expression was substantially attenuated following spermidine and cyclosporine treatment. These results suggest that PM_2.5_ exposure can be leads to pathological changes in the lacrimal gland, including inflammation, neovascularization and abnormal acini, due to CD4^+^ T cell immune responses and infiltration of CD3^+^ T lymphocyte and F4/80+ macrophages. Nevertheless, topical application of spermidine markedly suppressed these alterations of the lacrimal gland by PM_2.5_, and the efficacy of spermidine was similar to that of cyclosporine treatment.

**Figure 5:**
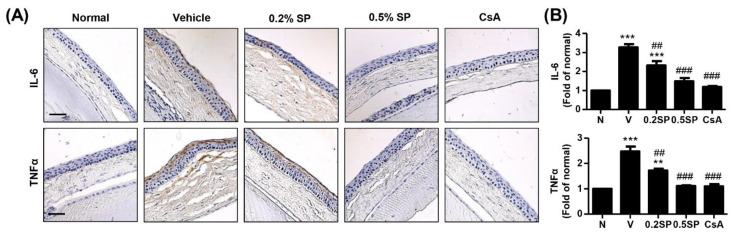
Effect of spermidine on inflammation of the cornea in a rat model of PM_2.5_-induced DED. (**A**) Representative images of immunohistochemical staining for IL-6 and TNF-α corneal sections. Scale bar; 50 μm. (**B**) The stained area of the photograph was quantitative analyzed using ImageJ^®^ and calculated in terms of the fold of the normal. The data are expressed as the means ± standard deviation (n = 3). ** *p* < 0.01 and *** *p* < 0.001 compared to normal group. ^##^ *p* < 0.01 and ^###^ *p* < 0.001 compared to the vehicle group.

In addition, we investigated whether PM_2.5_ involved in corneal inflammation that a critical role in the development of DED. Figure 5 showed that the expression of pro-inflammatory cytokines including IL-6 and TNF-α was significantly enhanced on corneal epithelium and stroma following by PM_2.5_ exposure, whereas its up-regulation was markedly reduced by spermidine and cyclosporine treatments.

### 3.7. Effect of Spermidine on Histological Changes of the Retina after Topical Exposure to PM_2.5_ in SD Rats

**Figure 6:**
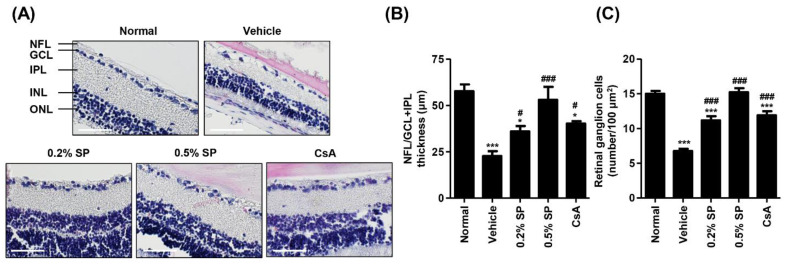
Effect of spermidine on retinal ganglion cell loss after topical exposure to PM_2.5_ in SD rats. (**A**) Representative images of cross-sectioned retina with H&E staining. Nerve fiber layer (NFL), ganglion cell layer (GCL), inner plexiform layer (IPL), inner nuclear layer (INL) and outer nuclear layer (ONL) are indicated. Scale bar; 50 μm. (**B**) Thickness of NFL/GCL +IPL layers. (**C**) The numbers of cells in GCL of 100 μm^2^ in five different sections were counted. Each bar indicates mean ± standard deviation (*n* = 5). The statistical analyses were conducted using analysis of ANOVA-Tukey’s post hoc test between groups. * *p* < 0.05 and *** *p* < 0.001 compared to the normal group. ^#^ *p* < 0.05 and ^###^ *p* < 0.001 compared to the vehicle group.

Next, we investigated the harmful effects on the retina following topical exposure to PM_2.5_, and the efficacy of spermidine on PM_2.5_-mediated retinal alteration. As a result of H&E staining in the retinal section, the thickness of the nerve fiber layer (NFL), ganglion cell layer (GCL) and inner plexiform layer (IPL) and inner plexiform layer markedly decreased by PM_2.5_, but this was markedly improved following 0.5% spermidine administration (Figure 6A,B). Meanwhile, the thickness of the inner nuclear layer (INL), and outer nuclear layer (ONL), did not differ among the groups. In addition, the population of ganglion cells in GCL was markedly decreased by exposure to topical PM_2.5_ (Figure 6A). However, the PM_2.5_-mediated decrease in ganglion cells was greatly improved after spermidine treatment. Figure 6C showed that the number of ganglion cells was significantly decreased in the retina of the vehicle group (7.50 ± 1.29/100 μm^2^) compared with that in the retina of the normal group (14.00 ± 1.41/100 μm^2^). In contrast, topical administration of 0.2% and 0.5% spermidine significantly improved the ganglion cell population to 11.50 ± 1.25/100 μm^2^ and 15.50 ± 1.30/100 μm^2^, respectively. The efficacy of 0.5% spermidine on the improvement of ganglion cells was superior to that of cyclosporine, similar to that of the 0.2% spermidine treatment.

## 4. Discussion

The ocular surface is composed of the corneal and conjunctival epithelium, corneoscleral limbus, nerves and tear film [30]. The ocular surface serves as a barrier to chemicals, microbes and other airborne matter and provides anatomic, physiologic and immunologic protective functions [30,31]. Therefore, the ocular surface could be an accessible model to study the direct influence of air pollution on human health [31]. Increasing epidemiological and biochemical evidence suggests that exposure to PM_2.5_ may be attributed to various ocular surface disorders, including DED, keratitis and conjunctivitis [9,10,11,12,13,14]. DED, also known as keratoconjunctivitis sicca, is a multifactorial disease of the ocular surface that involves tear film instability, hyperosmolarity, inflammation and damage to the ocular surface [32,33]. In addition, DED is caused by irritation, redness, stinging, pain and burning sensation and is involved in a decline in health-related quality of life [34]. Several animal studies have demonstrated that topical administration of PM_2.5_ presented dry eye phenotypes, accompanied by a decreased tear production, damaged corneal epithelium, reduced conjunctival goblet cells and an abnormal corneal structure [8,12,13,28]. In the present study, we also reproduced a DED murine model through topical instillation of PM_2.5_, which was accompanied by decreased tear secretion, induced detachment of the corneal epithelium and loss of conjunctival goblet cells, whereas spermidine eye drops improved tear production and stabilized the ocular surface.

The tear film is composed of three distinct layers and performs many physiological functions [35]. A mucin layer composed of mucins produced by conjunctival epithelial cells provides an easily wettable ocular surface and assists in tear re-spreading after blinking [36,37]. An aqueous layer conducts lubrication, prevents ocular surface dehydration, protects against pathogens and airborne substances, and supplies nutrients to the corneal epithelium [38]. A lipid layer reduces the surface tension of the tear film and protects against tear evaporation [35]. Therefore, compromise of the tear film triggers tear film destabilization, promotes exposure of the corneal epithelium to air and potentially contributes to dry eye symptoms [38]. Indeed, the initial step in the therapy of DED is focused on restoring the tear volume and compensating the tear components [39]. In this respect, our findings suggest that spermidine eye drops improve tear secretion and restore corneal epithelium damage in PM_2.5_-exposed eyes. According to the results of PAS staining of the conjunctiva, topical spermidine also enhanced the population of goblet cells. The conjunctival epithelium houses mucin-producing goblet cells and immune defense dendritic Langerhans cells [40]. Therefore, recovery of conjunctival goblet cells by spermidine dye drops may contribute to the stabilization of the mucin layer and lead to tear film stability. This finding supported that topical application of spermidine increased tear production and improved tear film stability through reinforcement of the mucin layer and was ultimately caused by ocular surface stability. CsA, used as a positive control in the current study, is an anti-inflammatory and a T cell immunomodulatory agent that is used to suppress ocular surface inflammation and improve tear film dynamics [41,42]. To date, the effect of CsA on PM_2.5_-exposed eyes is yet to be reported. Our results suggest that CsA attenuated the dry eye symptoms, including decreasing tear secretion, increasing CFS, epithelium detachment and loss of conjunctival goblet cells following topical exposure to PM_2.5_. However, this efficacy is less than that of spermidine.

The pathogenesis of DED involves a vicious cycle of inflammation [43]. Numerous evidence for inflammation in DED have been well-documented, including enhanced infiltration of immune cells in the conjunctiva and lacrimal glands, increased density of dendritic cells in the cornea and increased secretion levels of tear cytokines [42,43,44,45]. Inflammation of the ocular surface in DED is sustained by the ongoing activation and infiltration of pathogenic immune cells, primarily of CD4^+^ T cells [46]. Niederkorn et al. [47] identified CD4^+^ T cells from mice with DED inoculated with T cell-deficient mice, which led to inflammation in the lacrimal gland, cornea, conjunctiva and dry eye phenotype. In addition, it has been demonstrated that T helper 17 is concerned with the dysfunction of T regulatory cells and pathogenesis of DED [48]. Importantly, the main proliferating subset of dry eye-T cell effectors in the presence of T regulatory cells is IL-17 secreting CD4^+^ T cells [49]. IL-17 induces the secretion of pro-inflammatory cytokines such as IL-1, IL-6 and TNF-α, and these cytokines are upregulated in DED [50]. Lee et al. [51] suggested that the topical application of PM_2.5_ elevated dendritic cell maturation and the expression of TNF-α, IL-1β and IL-6 in a murine model. Hyun et al. [52] also reported that the expression of TNF-α and IL-6 was increased in the PM_2.5_-induced DED rat model, but extract of apricot kernel, the seed of *Prunus armeniaca* L. (Prunus, apricot), had a protective effect against DED by PM_2.5_. Even though the role of CD4^+^ T cell-mediated immune responses in DED is well-established, there are no studies on the relationship between CD4^+^ T cell-mediated immune responses in PM_2.5_-induced DED. One study verified that PM_2.5_-stimulated CD4^+^ T cells potently increased the mRNA and protein levels of pro-inflammatory cytokines and induced the death of human bronchial epithelial cells [53]. In the present study, we found that topical exposure to PM_2.5_ was caused by the pathological changes in the lacrimal gland, including inflammation, neovascularization and abnormal acini. Furthermore, PM_2.5_ increased the infiltration of CD3^+^ T lymphocytes, CD4^+^ T lymphocytes and F4/80+ macrophages, as well as the expression of pro-inflammatory cytokines including IL-17, and TNF-α in the lacrimal gland. In addition, our finding showed that topical exposure to PM_2.5_ enhanced the expression of pro-inflammatory cytokines such as IL-6 and TNF-α on the cornea. However, topical application of spermidine markedly suppressed these alterations of the lacrimal gland and cornea by PM_2.5_, and the efficacy of spermidine was similar to that of cyclosporine treatment. Even though these results suggest that spermidine eye drops may contribute to the suppression of CD4^+^ T cell immune responses induced by PM_2.5_. In addition, the suppressive effect of spermidine on lacrimal gland inflammation in PM_2.5_-exposed rats may due to its anti-inflammatory potential [22,23]. Nevertheless, we considered that further studies are needed to verify which subpopulation of lymphocytes involved in lacrimal gland pathogenesis, and which infiltrating cells release pro-inflammatory cytokines on the lacrimal gland.

Meanwhile, it has been reported that PM_2.5_ triggers systemic changes in the blood and contributes to various systemic diseases [54]. In addition, PM_2.5_ can destroy the integrity of the blood-brain barrier; thus, peripheral systemic inflammation easily crosses the barrier and influences the central nervous system [55]. Similarly, the blood-retina barrier (BRB) is available for diffusion and permeabilization of PM_2.5_, and its function in BRB could potentially play a role in PM_2.5_-mediated retinal pathogenesis [7,56,57,58,59,60]. Several epidemiological studies have suggested that ambient PM_2.5_ result in increasing prevalence rates of retinal disorders including age-related macular degeneration, diabetic retinopathy and sclerosis of the retina [59,61,62,63]. Furthermore, a few recent studies demonstrated that PM_2.5_ exposure involved in retinal disorders including retinal damage, including retinal atherosclerosis, retinal edema and retinal vessel narrowing [57,58,59]. Kang et al. [7] demonstrated that topical exposure to PM_2.5_ markedly decreased the NFL/GCL and increased the expression of glial fibrillary acidic protein, a marker of glial activation in response to neural injury [60]. Furthermore, Chua et al. [57] reported that subjects exposed to higher levels of PM_2.5_ were associated with changes in retinal structure features, including RCG and IPL thickness. Previously, we suggested that PM_2.5_ mediates retinal dysfunction through ROS-mediated epithelial-mesenchymal transition and necrotic and autophagic cell death in retinal pigment epithelial cells [64,65]. Additionally, our current findings showed that topical exposure to PM_2.5_ led to the loss of retinal ganglion cells and the decrease of the NFL/GCL+IPL thickness; however, spermidine eye drops contribute to the normalization of retinal ganglion cell density and retinal structure. In this regard, our present finding was very meaningful in that topical exposure to PM_2.5_ mediated harmful influence on the posterior eye segment, such as RGC loss and alteration of retinal layer composition. Additionally, our findings are consistent with those of previous reports that spermidine has a protective effect on ganglion cell death in an optic nerve injury mouse and autoimmune encephalomyelitis mice [21,25]. In this sense, our finding suggested that topical application of spermidine may have not only protective effect on PM_2.5_-induced DED symptoms, but also improve effect on PM_2.5_-mediated retinal disorders.

In the present study, we also investigated the effect of spermidine eye drops on the changes of the eyes, as well as on the hematology and biochemistry in the topical exposure of PM_2.5_-induced DED rats. Interestingly, topical exposure to PM_2.5_ led to partial abnormality of the serum lipid profile, including TC and LDL-C. However, our findings showed that spermidine eye drops contribute to the normalization of TC and LDL-C levels. Increasing epidemiological studies have recently shown that exposure to ambient PM_2.5_ may have a hazardous influence on the metabolic system, including an increased risk of dyslipidemia [63,64]. One nationwide cohort study demonstrated that exposure to ambient PM_2.5_ is associated with worsening LDL-C levels [66]. Another cohort study suggested that high levels of fine PM exposure were closely correlated with increasing TC and LDL-C levels [67]. Even though these few studies reported that exposure to ambient PM_2.5_ may have a negative effect on lipid profiles, such as TC and LDL-C, no studies have investigated the influence of topical exposure to PM_2.5_ on serum lipid profiles. In this context, our findings were very meaningful in that the levels of serum TC and LDL-C were markedly increased by topical exposure to PM_2.5_, but this change in the lipid profile was decreased by spermidine treatment.

## 5. Conclusions

Taken together, our current results showed that topical spermidine attenuated tear film destabilization, inflammation of the lacrimal gland and histological changes in the retinal GCL and IPL in the PM_2.5_-induced DED rat model. Therefore, the present findings may provide an experimental basis for the potential application of spermidine in treating air pollution-related dry eye symptoms and retinal disorders.

## Figures and Tables

**Table 1:** Changes in the weight of organs after 2 weeks of treatment in PM_2.5_-topical exposed Sprague-Dawley rats.

Organ Weight (g)	Group				
	Normal	Vehicle	0.2% SP	0.5% SP	CsA
Body weight gain	32.53 ± 11.91	26.20 ± 8.23	28.34 ± 8.37	31.35 ± 9.09	36.17 ± 14.19
Thymus	0.41 ± 0.06	0.38 ± 0.10	0.37 ± 0.09	0.43 ± 0.10	0.39 ± 0.10
Heart	0.72 ± 0.01	0.68 ± 0.02	0.67 ± 0.03	0.70 ± 0.04	0.68 ± 0.07
Lung	1.10 ± 0.07	1.04 ± 0.06	1.00 ±0.04	1.02 ± 0.07	1.02 ± 0.06
Liver	6.58 ± 0.53	6.57 ± 0.24	6.17 ± 0.72	6.57 ± 0.36	6.59 ± 0.52
Kidney	1.57 ± 0.08	1.50 ± 0.03	1.46 ± 0.20	1.45 ± 0.11	1.51 ± 0.16
Spleen	0.53 ± 0.08	0.52 ± 0.06	0.58 ± 0.08	0.51 ± 0.05	0.55 ± 0.05

SD rats were sacrificed on day 14 after the treatment. The thymus, heart, lung, liver, kidney, spleen, uterus and ovaries were immediately excised and weighed. The data were expressed as mean ± standard deviation (*n* = 5). Statistical analyses were conducted using analysis of ANOVA-Tukey’s post hoc test between groups. All data showed that there were no statistically significant differences between groups. Normal, untreated normal control group; vehicle, PM_2.5_ with normal saline-treated group; 0.2% SP, PM_2.5_ with 0.2% spermidine-treated group; 0.5% SP, PM_2.5_ with 0.5% spermidine-treated group; and CsA, PM_2.5_ with cyclosporine A-treated group. CsA, cyclosporin A.

**Table 2:** Changes in the hematological, biochemical and lipid profiles after 2 weeks of treatment in PM_2.5_-topical exposed SD rats.

Parameter (Units)	Group				
	Normal	Vehicle	0.2% SP	0.5% SP	CsA
RBC (10^6^/μL)	8.11 ± 0.32	8.25 ± 0.14	8.09 ± 0.27	7.96 ± 0.21	7.79 ± 0.36
WBC (10^3^/μL)	5.17 ± 0.64	4.98 ± 1.84	4.30 ± 0.80	4.13 ± 1.49	4.12 ± 1.73
Hematocrit (%)	49.86 ± 1.83	49.60 ± 0.75	48.26 ± 1.54	48.94 ± 2.02	48.48 ± 1.44
Hemoglobin (g/dL)	15.52 ± 0.18	15.42 ± 0.25	15.32 ± 0.48	14.92 ± 0.53	14.86 ± 0.36
MCV (fL)	61.54 ± 1.43	60.13 ± 0.63	59.66 ± 0.83	61.48 ± 1.39	62.34 ± 1.14
MCH (pg)	19.18 ± 0.62	18.68 ± 0.16	18.94 ± 0.28	18.72 ± 0.22	19.14 ± 0.67
Platelet (10^3^/μL)	791.40 ± 128.32	935.67 ± 88.50	899.20 ± 68.88	955.40 ± 79.64	943.80 ± 81.62
AST (U/L)	138.96 ± 27.49	152.58 ± 24.23	167.06 ± 34.21	157.98 ± 22.57	134.18 ± 25.11
ALT (U/L)	20.60 ± 3.18	23.50 ± 4.66	23.90 ± 3.80	24.50 ± 4.72	19.78 ± 3.20
ALP (U/L)	420.30 ± 41.41	422.85 ± 78.25	432.38 ± 66.61	454.80 ± 76.49	457.36 ± 117.58
BUN (mg/dL)	13.15 ± 1.89	13.81 ± 1.28	14.58 ± 0.04	14.69 ± 1.91	15.43 ± 3.13
Creatinine (mg/dL)	0.46 ± 0.02	0.47 ± 0.05	0.50 ± 0.04	0.50 ± 0.04	0.51 ± 0.04
TC (mg/dL)	58.25 ± 3.85	74.42 ± 6.77 *	65.43 ± 12.06	57.16 ± 6.71 ^#^	58.15 ± 6.88 ^#^
TG (mg/dL)	48.68 ± 18.05	46.43 ± 10.01	43.24 ± 9.17	36.36 ± 14.75	44.70 ± 9.98
HDL-C (mg/dL)	29.92 ± 5.47	30.58 ± 2.03	26.00 ± 4.68	25.12 ± 3.53	25.98 ± 1.19
LDL-C (mg/dL)	6.03 ± 1.36	9.62 ± 1.64 *	7.63 ± 2.26	6.75 ± 1.09	7.30 ± 1.71
FFA (uEq/L)	654.20 ± 46.55	725.50 ± 102.97	662.40 ± 21.17	612.20 ± 89.71	672.40 ± 101.21

On day 14 after treatment, whole blood was analyzed for hematological and biochemical evaluation. The data were expressed as mean ± standard deviation (*n* = 5). Statistical analyses were conducted using analysis of ANOVA-Tukey’s post hoc test between groups. * *p* < 0.05, compared to the normal group. ^#^ *p* < 0.05, compared to vehicle group. RBC, red blood cells; WBC, white blood cells; MCV, mean corpuscular volume; MCH, mean corpuscular hemoglobin; AST, aspartate aminotransferase; ALT, alanine aminotransferase; ALP, alkaline phosphatase; BUN, blood urea nitrogen; TC, total cholesterol; TG, triglyceride; HDL-C, high-density lipoprotein cholesterol; LDL, low-density lipoprotein cholesterol; FFA, free fatty acid.

## Data Availability

The datasets during and/or analyzed during the current study available from the corresponding author on reasonable request.
